# Denosumab Therapy in Dental Practice: Awareness, Risk Factors and Preventive Strategies

**DOI:** 10.1155/joos/9628549

**Published:** 2026-04-30

**Authors:** Kumara Kaluarachchi, Indika Kulatunga, Thanaphum Osathanon, Lakshman Samaranayake

**Affiliations:** ^1^ Department of Pharmacology, Faculty of Medicine and Allied Sciences, Rajarata University, Mihintale, Sri Lanka, rjt.ac.lk; ^2^ Army Hospital, Colombo, Sri Lanka; ^3^ Center of Excellence for Dental Stem Cell Biology and Department of Anatomy, Faculty of Dentistry, Chulalongkorn University, 34 Henri-Dunant Rd. Pathumwan, Bangkok, 10330, Thailand, chula.ac.th; ^4^ Center of Excellence in Periodontal Disease and Dental Implantology, Faculty of Dentistry, Chulalongkorn University, Bangkok, Thailand, chula.ac.th

**Keywords:** antiresorptive therapy, denosumab, dental management, medication-related osteonecrosis of the jaw (MRONJ), osteoporosis, risk factors

## Abstract

Denosumab is increasingly prescribed for osteoporosis and metastatic bone disease, leading to a parallel rise in reports of medication‐related osteonecrosis of the jaw (MRONJ). Although the condition was first associated with bisphosphonate therapy, emerging evidence indicates that denosumab‐associated MRONJ presents distinct epidemiological, clinical and management challenges. Despite an expanding body of research, awareness of MRONJ risk among general dental practitioners remains inconsistent, contributing to preventable adverse outcomes. This narrative review consolidates current knowledge on MRONJ in denosumab‐treated patients by examining epidemiology, pathophysiology, clinical features, established risk factors and evidence‐based preventive strategies. Numerous guidelines consistently emphasise early dental assessment, optimisation of oral health prior to the first denosumab dose and timely communication between physicians and dental clinicians. A planned dental intervention may be safer when coordinated around the denosumab dosing schedule, particularly by utilising a “window of opportunity” during periods of the waning drug effect. This review also synthesises practical recommendations for routine dental care, pretreatment evaluation, risk stratification and management of established MRONJ. A clinical decision‐making flowchart is proposed to assist dental practitioners in evaluating treatment needs, assessing patient risk and planning interventions with minimal disruption to systemic therapy. Taken together, understanding the unique characteristics of denosumab‐associated MRONJ is essential for reducing morbidity and improving patient outcomes. Strengthening collaboration between dental practitioners and medical prescribers, along with early identification of vulnerable patients, remains the cornerstone of prevention.

## 1. Introduction

Denosumab is a monoclonal antibody targeting receptor activator of nuclear factor‐κB ligand (RANKL). It has become a cornerstone therapy for the management of osteoporosis and the prevention of skeletal‐related events (SREs) in patients with metastatic bone disease [1]. Its potent but reversible inhibition of osteoclast activity offers significant therapeutic advantages over bisphosphonates, particularly in improving bone mineral density and reducing fracture risk [2]. However, accumulating evidence over the past decade has linked denosumab therapy with medication‐related osteonecrosis of the jaw (MRONJ), a serious adverse event characterised by the persistent exposed bone or the bone that can be probed through an intraoral or extraoral fistula, lasting more than 8 weeks in patients with no history of craniofacial radiotherapy [3].

While MRONJ has historically been associated with bisphosphonates, recent clinical studies indicate that the risk profile for denosumab is distinct. MRONJ can occur even at the lower doses used for osteoporosis, with incidence rates higher than originally anticipated. High‐dose regimens used in oncology settings carry an even greater risk [4]. Dental extractions, periodontal disease, peri‐implantitis and poor oral hygiene remain major triggers, but cases have also been reported in the absence of invasive dental procedures, highlighting potential differences in pathophysiology between denosumab and bisphosphonate‐associated lesions [5].

As the global population ages and denosumab prescriptions continue to rise, dental practitioners are increasingly likely to encounter patients receiving this therapy [1]. The dental implications are substantial: Treatment planning must account for altered bone turnover, surgical timing must be coordinated with dosing intervals and early recognition of MRONJ requires a high index of suspicion [2]. Despite this growing clinical need, awareness among dental professionals remains variable, and evidence‐based protocols are inconsistently applied in practice.

This review synthesises the current knowledge on denosumab‐associated MRONJ, encompassing epidemiology, risk factors, pathophysiological mechanisms, clinical presentation and preventive and therapeutic strategies. Particular emphasis is placed on practical guidance for dental practitioners, including a structured clinical workflow designed to support safe, evidence‐based decision‐making and optimise patient management. By strengthening the understanding of denosumab’s effects and standardising dental care protocols, clinicians can effectively minimise MRONJ risk and improve the outcomes for this growing patient population.

## 2. Denosumab Targets the RANK–RANKL Pathway

**FIGURE 1:**
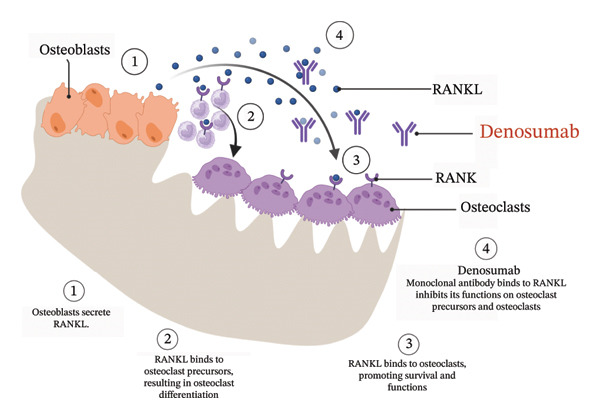
Schematic diagram illustrating the mechanism of action of denosumab through inhibition of the RANK–RANKL pathway. Schematic diagram illustrating the mechanism of action of denosumab. Denosumab binds with high affinity to receptor activator of nuclear factor‐κB ligand (RANKL), thereby preventing its interaction with the receptor activator of nuclear factor‐κB (RANK) on osteoclast precursors and mature osteoclasts. Inhibition of the RANK–RANKL signalling pathway suppresses osteoclast formation, function, and survival, resulting in reduced bone resorption and preservation of bone mass. Created in BioRender. https://BioRender.com/47ipl22.

Denosumab is a full human monoclonal antibody that binds with high specificity to RANKL, a cytokine essential for the differentiation, activation and survival of osteoclasts. Under normal physiology, RANKL expressed by osteoblasts and stromal cells interacts with its receptor RANK on osteoclast precursors, driving their differentiation into bone‐resorbing osteoclasts [3]. This RANK/RANKL signalling also sustains osteoclast activity and prolongs their lifespan, maintaining bone turnover [4]. By neutralising RANKL, denosumab prevents its binding to RANK, thereby suppressing osteoclast formation and function [5] (Figure 1). The result is a marked reduction in bone resorption, increased bone mineral density and protection against SREs in osteoporosis and cancer‐related bone disease. Beyond osteoclasts, the RANK–RANKL axis also influences immune cells such as dendritic cells and T lymphocytes, contributing to bone–immune system cross‐talk [6]. Denosumab’s blockade of RANKL may therefore have secondary effects on local immune responses and angiogenesis, which partly explain its association with complications such as MRONJ. In summary, denosumab exerts its therapeutic action by interrupting the osteoclastogenic cascade at the RANK–RANKL interface, profoundly altering bone remodelling dynamics.

## 3. Epidemiology of Denosumab‐Associated MRONJ

MRONJ is increasingly recognised as a clinically significant adverse effect of antiresorptive therapies, including denosumab. Epidemiological data indicate that the incidence of MRONJ varies substantially depending on the patient population, dose regimen, duration of therapy and underlying comorbidities [7]. In patients receiving denosumab for osteoporosis (Prolia, 60 mg every 6 months), the reported incidence is low, ranging from 0.04% to 0.3%, reflecting the relatively lower cumulative exposure and the absence of concurrent oncologic risk factors. Conversely, among oncology patients receiving high‐dose denosumab (Xgeva, 120 mg monthly) for the prevention of SREs, incidence rates are considerably higher, reported between 1% and 9% in large retrospective cohorts and clinical trials [8].

Several population‐based studies have explored the distribution of MRONJ cases across age, sex and geographic location. The majority of affected patients are older adults, consistent with the primary indications for osteoporosis and metastatic bone diseases. Both sexes are affected, although some studies suggest a slightly higher prevalence in women, reflecting the higher proportion of postmenopausal patients receiving osteoporosis therapy [9].

Longitudinal studies indicate that the cumulative risk of MRONJ increases with therapy duration, particularly beyond 2 years of continuous denosumab administration. Additional cofactors such as diabetes mellitus, corticosteroid use, poor oral hygiene [10], and history of invasive dental procedures further elevate risk. Notably, cases of MRONJ have been documented even in the absence of dental extractions or trauma, suggesting that intrinsic alterations in bone turnover and local microenvironment may contribute to pathogenesis in denosumab‐treated patients [11].

Although precise incidence rates vary across studies, the consistent observation is that MRONJ, while uncommon in osteoporosis therapy, is a clinically important risk in oncology populations. Understanding these epidemiological patterns is critical for dental practitioners to identify high‐risk patients, implement preventive strategies, and optimise procedural timing to mitigate adverse outcomes.

## 4. Denosumab as a Biologic Adjunct in Oral and Maxillofacial Surgery (OMF)

Denosumab is increasingly used by OMF surgeons as a targeted therapy for giant cell tumours and central giant cell granulomas (CGCGs) of the jaws, especially in cases where surgery alone is difficult or carries high morbidity [12]. For OMF surgeons, this biologic agent offers several advantages:

a. Adjunct to surgery: Denosumab can shrink or stabilise lesions before surgical intervention, making resections less extensive and sometimes avoiding radical procedures.
b. Management of aggressive jaw lesions: CGCGs of the jaws, which can be locally destructive, have shown a good response to denosumab therapy.
c. Improved functional outcomes: By reducing lesion size, surgeons can preserve more bone and teeth, maintaining oral function and aesthetics.
d. Alternative in complex cases: In patients with recurrent or unresectable lesions, denosumab provides a nonsurgical option that still achieves disease control.
e. Need for monitoring: OMF surgeons must carefully monitor for side effects such as hypocalcemia and osteonecrosis of the jaw, which are particularly relevant in dental and maxillofacial contexts.

Recent reviews and case series in the OMF surgery literature highlight denosumab’s effectiveness in treating CGCG of the jaws, with reports of significant lesion regression and improved surgical feasibility [13]. However, long‐term outcomes and recurrence rates after discontinuation remain areas of the ongoing study.

## 5. Pathophysiology of Denosumab‐Associated MRONJ

Denosumab effectively suppresses osteoclast‐mediated bone resorption. This antiresorptive action underpins its therapeutic efficacy in osteoporosis and metastatic bone disease, resulting in increased bone mineral density and reduced SREs [3]. However, the same mechanism is central to the development of MRONJ.

The jawbone is particularly susceptible to osteonecrosis due to its high turnover rate, unique vascular anatomy and frequent exposure to microtrauma from mastication and dental procedures [14]. Denosumab‐induced suppression of osteoclast activity disrupts the normal bone remodelling cycle, leading to accumulation of microdamage and impaired healing capacity [15]. Consequently, even minor oral injuries or surgical interventions can precipitate necrosis. Unlike bisphosphonates, which bind to hydroxyapatite and persist in bone for years, denosumab’s effect is reversible, with osteoclast function typically recovering within 6–9 months after cessation [16]. Despite this, MRONJ can still occur during therapy, particularly when bone turnover suppression coincides with trauma or infection [7].

Additional pathophysiological factors contribute to the development of MRONJ. Denosumab may alter local immune responses, impair angiogenesis and reduce the bone’s ability to combat bacterial colonisation, facilitating the persistence of infection and necrosis [17]. Coexisting risk factors, including corticosteroid therapy, diabetes, chemotherapy, poor oral hygiene and invasive dental procedures, further exacerbate these effects [10]. The interplay between systemic antiresorptive therapy and local oral conditions thus creates a “perfect storm” for MRONJ development, distinguishing denosumab‐associated lesions from those related to bisphosphonates in onset, severity and healing potential [8].

Understanding the RANKL–osteoclast axis and its consequences for jawbone physiology is essential for dental practitioners. It highlights the importance of preventive measures, careful surgical planning and timely coordination with prescribing physicians to mitigate MRONJ risk while maintaining therapeutic benefit from denosumab.

## 6. Risk Factors for Denosumab‐Associated MRONJ

The development of MRONJ in denosumab‐treated patients is multifactorial, arising from the interplay of systemic, local and treatment‐related factors [18]. Identifying these risk determinants is essential for dental practitioners to stratify patients, guide preventive strategies and optimise clinical outcomes.

### 6.1. Medication‐Related Factors

The dosage and indication of denosumab are primary determinants of MRONJ risk. High‐dose regimens, such as Xgeva (120 mg monthly), used in oncology for metastatic bone disease, are associated with higher incidence rates compared to low‐dose regimens (Prolia, 60 mg every 6 months) prescribed for osteoporosis [1, 19]. Longer cumulative exposure increases the likelihood of MRONJ, particularly beyond 2 years of therapy [7]. Concurrent use of corticosteroids, chemotherapy or other antiresorptive agents further elevates the risk by compounding immunosuppression and impairing bone healing [10].

### 6.2. Dental Procedure–Related Factors

Invasive dental procedures, especially tooth extractions, apical surgery, periodontal flap surgery and implant placement, are consistently identified as major triggers for MRONJ [20]. Extractions are particularly high‐risk due to direct disruption of bone integrity in an environment of suppressed remodelling [21]. Even minor surgical interventions or poorly executed restorations can precipitate lesions in susceptible patients. Conversely, noninvasive procedures such as scaling, fillings and endodontic therapy generally carry minimal risk [22].

### 6.3. Local Oral Conditions

Periodontal disease, poor oral hygiene, ill‐fitting dentures, chronic periapical infections and peri‐implantitis create a proinflammatory microenvironment that predisposes the jawbone to necrosis [1]. The mandible is more frequently affected than the maxilla, likely due to relatively lower vascularity and higher mechanical stress [14].

### 6.4. Patient‐Related Factors

Advanced age, diabetes mellitus, immunocompromised status and smoking have been identified as independent risk modifiers [1, 10, 11, 17]. These factors impair wound healing and local immune response, increasing susceptibility to necrosis following even minor trauma. Additionally, genetic predispositions and variations in bone metabolism may play a role, though these remain under investigation [23].

Denosumab‐associated MRONJ is most likely to occur at the intersection of high‐dose or prolonged therapy, invasive dental procedures, compromised oral health and systemic patient vulnerabilities16. Comprehensive risk assessment before initiating denosumab therapy or performing dental interventions is crucial to prevent MRONJ and ensure safe patient care.

## 7. The “Drug Holiday” Debate

Recent clinical evidence indicates that the traditional concept of a “drug holiday”—a planned interruption in treatment to minimize long‐term side effects—is not appropriate for denosumab. Unlike medications that remain in the bone matrix for extended periods, the biological effects of denosumab are rapidly reversible [24]. In the context of solid cancer bone metastasis, extending dosing intervals may be feasible in the short term, but actual discontinuation leads to a significant rise in SREs approximately 25 weeks after the final dose [25].

This “rebound effect” is characterized by a surge in bone turnover markers and a precipitous decline in bone mineral density. In osteoporosis management, this rebound can result in a high risk of spontaneous multiple vertebral fractures if the cessation of denosumab is not immediately managed with sequential therapy [24]. Furthermore, longitudinal data suggest that maintaining a continuous 10‐year regimen is more cost‐effective than strategies involving treatment interruptions, as the clinical and economic costs of fractures following a “holiday” far outweigh the savings of pausing the medication [26]. Consequently, denosumab should only be stopped if it is immediately followed by a “relay” treatment, such as a bisphosphonate, to maintain skeletal integrity and prevent the rapid loss of treatment gains [24, 25].

## 8. Dental Procedures and Clinical Management in Denosumab‐Treated Patients

Dental practitioners play a pivotal role in mitigating the risk of MRONJ among patients receiving denosumab. Effective management strategies must be tailored to the specific invasiveness of the dental procedure, the patient’s underlying medical risk profile, and the precise timing relative to denosumab administration.

For low‐risk procedures, which include noninvasive or minimally invasive interventions, the risk of developing MRONJ is generally considered minimal. Such procedures typically do not require any alteration to the denosumab therapy regimen. This category encompasses routine dental examinations, prophylaxis such as scaling and polishing, restorative treatments including fillings and crowns, and endodontic therapy that does not involve periapical surgery. Additionally, orthodontic adjustments and simple atraumatic procedures are considered safe. Throughout these treatments, maintaining excellent oral hygiene and adhering to routine preventive care remain critical objectives to reduce the eventual necessity for more invasive interventions.

Procedures classified as carrying a moderate risk involve limited bone manipulation or minor surgical intervention. This intermediate risk category includes deep periodontal scaling, root planning, minor soft tissue surgeries, and conservative periapical surgery. When performing these tasks, practitioners must ensure meticulous asepsis and utilize techniques that minimize tissue trauma. For patients being treated for osteoporosis, it is often advisable to time these moderate‐risk procedures toward the end of the dosing interval to align with the waning pharmacological effect of the medication.

Invasive procedures characterized by direct bone exposure carry the highest risk for the development of MRONJ. These high‐risk interventions include simple or surgical tooth extractions—particularly when multiple teeth are involved—dental implant placement, peri‐implant bone grafting, apical surgery, periodontal flap surgery and complex oral surgeries or osteotomies. Whenever feasible, high‐risk procedures should be planned through deliberate interprofessional coordination between the dentist and the prescribing physician. In the context of osteoporosis treatment, a “window of opportunity” for these surgeries typically occurs approximately five to 6 months after the most recent injection. Conversely, oncology patients receiving more frequent, higher‐dose denosumab regimens face a significantly elevated risk profile due to the shorter dosing intervals, necessitating even more stringent preventive strategies and multidisciplinary oversight.

## 9. Preventive Strategies for the Mitigation of MRONJ

The prevention of MRONJ is a multidisciplinary responsibility that begins prior to the initiation of denosumab therapy. A comprehensive baseline dental assessment is the cornerstone of prevention, allowing for the identification and elimination of potential sources of infection before bone remodelling is pharmacologically altered. Ideally, all necessary invasive dental procedures, including the resolution of active caries and the extraction of nonrestorable teeth, should be completed, and the sites allowed to heal before the first dose of denosumab is administered. This proactive approach significantly reduces the need for high‐risk surgical interventions during the active treatment phase.

Beyond initial clearance, the ongoing optimization of periodontal health is essential. Chronic periodontal disease serves as a persistent inflammatory stimulus that increases the risk of bone necrosis; therefore, maintaining a stable oral environment through regular professional monitoring is vital. Patient education serves as a critical secondary preventive measure, ensuring that individuals understand the importance of meticulous oral hygiene and the necessity of reporting early symptoms, such as jaw pain, swelling, or exposed bone, immediately. When dental procedures become necessary during active denosumab therapy, practitioners should employ clinical protocols designed to minimize trauma. This includes the use of minimally invasive surgical techniques, the application of local antiseptics such as chlorhexidine, and the judicious use of systemic antibiotic prophylaxis in cases where the risk of postoperative infection is high. By integrating these preventive layers, the clinical team can significantly lower the incidence of MRONJ and maintain the patient’s quality of life throughout their treatment course.

## 10. Multidisciplinary Integration in the Management of Patients Receiving Denosumab

The clinical management of patients undergoing denosumab therapy necessitates a robust multidisciplinary framework to navigate the complex intersection of systemic bone health and localized oral complications. Effective care is predicated on seamless collaboration between dental practitioners, oncologists, endocrinologists and OMF surgeons. This integrated model ensures that treatment decisions are not made in isolation but are instead balanced to prioritize both the management of the primary condition—whether it be osteoporosis or SREs in oncology—and the preservation of maxillofacial integrity. By establishing clear lines of communication, the care team can synchronize the timing of dental interventions with the medication’s pharmacological profile, thereby optimizing the “window of opportunity” for necessary surgeries and reducing the cumulative risk of MRONJ [27].

Utilizing multidisciplinary language within clinical protocols underscores the shared responsibility across medical and dental specialties and highlights dentistry’s integral role in the broader landscape of comprehensive patient care. In this model, the dental surgeon serves not only as a clinician but as a vital consultant to the oncology or metabolic team, providing critical risk assessments that inform the systemic treatment plan. For instance, in complex cases where dental health is compromised, the multidisciplinary team may collectively decide to delay the initiation of denosumab or adjust the dosing schedule to allow for adequate oral healing. Furthermore, the involvement of maxillofacial specialists in the early stages of bone‐modifying therapy ensures that any emergent symptoms are addressed with expert surgical oversight. Ultimately, this collaborative approach transcends traditional specialty silos, fostering a patient‐centred environment where therapeutic efficacy is maximized and the risk of debilitating oral complications is minimized.

## 11. Clinical Workflow

**FIGURE 2:**
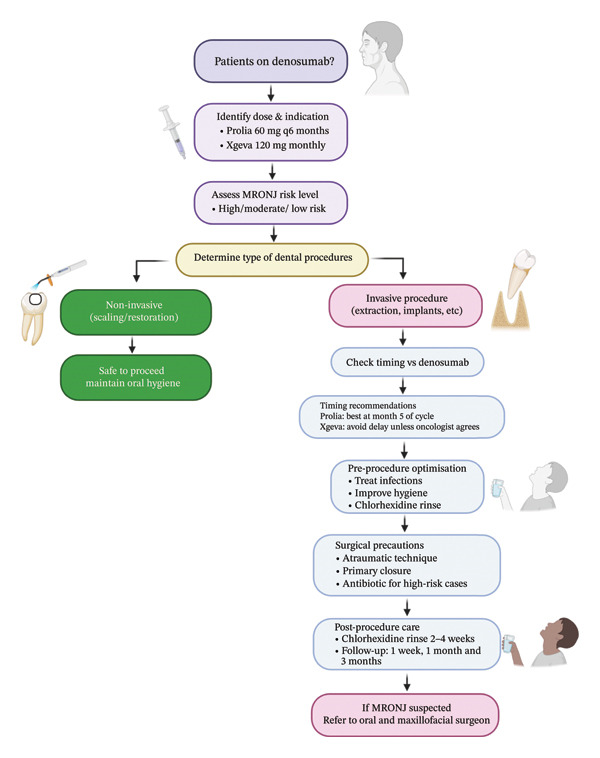
Schematic diagram illustrating the clinical workflow for safely managing patients receiving denosumab therapy. A structured clinical approach guiding dentists in the safe management of patients receiving denosumab therapy. Initial assessment includes evaluation of systemic and oral risk factors, allowing stratification into low, moderate or high risk for complications and enabling tailored preventive strategies. Preventive measures encompass pretreatment dental optimisation, reinforcement of oral hygiene practices and patient education on early warning signs. Procedural planning aims to minimise trauma, appropriately time interventions in relation to the denosumab dosing cycle, and facilitate coordination with oral and maxillofacial surgeons when required. Continuous monitoring for early features of medication‐related osteonecrosis of the jaw (MRONJ), including exposed bone, delayed healing or unexplained pain, is essential for timely intervention. Patients with advanced lesions should be promptly referred to specialists, with ongoing multidisciplinary collaboration ensuring adaptive care and optimised patient safety throughout therapy. Created in BioRender https://BioRender.com/47ipl22.

A structured clinical approach can guide dentists in safely managing patients receiving denosumab therapy (Figure 2). Initial patient assessment should include evaluation of systemic and oral risk factors. Based on this, patients can be stratified into low, moderate or high risk for complications, enabling tailored preventive strategies [28]. Preventive measures should include pretreatment dental optimisation, reinforcement of oral hygiene practices and patient education regarding early warning signs. Procedural planning should aim to minimise trauma, schedule interventions appropriately relative to the denosumab dosing cycle and involve coordination with oral and maxillofacial surgeons when necessary. Continuous monitoring for early signs of MRONJ, such as exposed bone, delayed healing, or unexplained pain, is essential for timely intervention. Patients presenting with advanced lesions should be promptly referred to specialists, while ongoing collaboration between dentists, surgeons and prescribing physicians ensures adaptive care and optimises patient safety throughout therapy.

Through this integrated workflow, dentists can proactively mitigate risk, provide safe and evidence‐based care and reduce the incidence and severity of complications in patients receiving denosumab therapy. This structured approach shifts focus from reactive treatment of MRONJ to proactive prevention and early intervention, improving clinical outcomes and patient safety.

## 12. Management of Established MRONJ

Management of established MRONJ primarily falls under the expertise of OMF surgeons, although dental practitioners play a key supportive and referral role. The approach depends on disease stage, severity, symptoms and patient comorbidities.

### 12.1. Staging and Assessment

MRONJ is commonly staged according to the American Association of Oral and Maxillofacial Surgeons (AAOMS) criteria [29].

a. Stage 0: No clinical evidence of necrotic bone, but nonspecific symptoms or radiographic changes
b. Stage 1: Exposed necrotic bone without infection or symptoms
c. Stage 2: Exposed necrotic bone with pain and infection
d. Stage 3: Advanced disease with pathologic fracture, extraoral fistula or osteolysis extending to the inferior border

Accurate staging guides treatment planning and helps determine when surgical intervention is warranted.

### 12.2. Conservative Management (Dentist‐Led Supportive Care)

Stage 0 can be managed by general dental practitioners, but Stage 1 should be referred to, or at least treated, in liaison with an OMFS. For early‐stage or asymptomatic cases, conservative measures aim to prevent progression and relieve symptoms:

a. Antimicrobial mouth rinses (e.g., chlorhexidine)
b. Systemic antibiotics for secondary infections
c. Analgesics for pain control
d. Regular monitoring and professional dental cleaning
e. Avoidance of further invasive procedures

Dental practitioners are responsible for early recognition, monitoring disease progression and maintaining oral hygiene5.

### 12.3. Surgical Management (OMF Surgeon‐Led)

Definitive intervention is indicated for

a. Stage 2 patients with persistent infection or pain unresponsive to conservative care
b. Stage 3 patients with extensive necrosis, pathological fracture or fistula formation

Surgical options include debridement, sequestrectomy, marginal resection or segmental resection, depending on severity. Surgery is ideally performed with minimal trauma, using atraumatic techniques and primary closure where possible to facilitate healing. Emerging evidence suggests that adjunctive therapies such as platelet‐rich plasma (PRP), pentoxifylline and hyperbaric oxygen may offer additional benefit in selected cases.

### 12.4. Interprofessional Collaboration

Optimal outcomes require coordinated care between dentists, OMFS surgeons and the prescribing physician. Dentists ensure ongoing oral hygiene, monitor for recurrence and manage minor complications, while OMFS surgeons handle complex surgical interventions. Communication regarding denosumab dosing schedules and patient comorbidities is essential to minimise risk and maximise healing.

### 12.5. Follow‐Up and Long‐Term Care

Posttreatment follow‐up is critical, involving regular clinical and radiographic evaluation to detect residual necrotic bone or recurrent lesions. Preventive dental care and patient education remain central to reducing future MRONJ risk.

A structured clinical approach enables dental practitioners to mitigate risk and deliver safe care to denosumab‐treated patients. The initial assessment should encompass the denosumab dosing history, systemic and oral health evaluations, and identification of risk factors. Patients can then be stratified according to risk level, guiding the extent and timing of interventions. Preventive care, procedural planning, early detection and timely referral constitute the core components of risk reduction. Regular monitoring and interprofessional communication allow adaptive management, accommodating changes in therapy, patient health or oral status. Integrating these strategies into routine dental practice shifts focus from reactive management to proactive prevention, reducing complications and enhancing patient safety.

## 13. Discussion

Denosumab represents a paradigm shift in antiresorptive therapy, offering potent efficacy in fracture prevention and skeletal protection. However, the suppression of bone remodelling introduces significant dental considerations. The relatively low incidence of MRONJ in osteoporosis populations belies its potentially severe consequences, especially among oncology patients or those undergoing invasive procedures. Dental practitioners are therefore positioned to significantly impact outcomes through preventive strategies, early detection, and interprofessional collaboration. By understanding denosumab’s pharmacology, patient‐specific risk factors, and the timing of interventions, dentists can minimise complications and support safe continuation of therapy. Emphasising prevention over reactive treatment ensures patient safety and optimises clinical outcomes.

## 14. Conclusion

Denosumab therapy alters bone remodelling, increasing susceptibility to jaw complications such as MRONJ. Dental practitioners play a critical role in risk reduction through early assessment, preventive interventions, patient education and monitoring. Awareness of pharmacology, dosing schedules and risk factors enables safe procedural planning, timely referral and interprofessional coordination. Integrating denosumab awareness into dental practice ensures that patients benefit from therapy while minimising the risk of serious complications.

## Funding

This research did not receive any specific grant from funding agencies in the public, commercial or not‐for‐profit sectors.

## Conflicts of Interest

The authors declare no conflicts of interest.

## Data Availability

The data that support the findings of this study are available on request from the corresponding author. The data are not publicly available due to privacy or ethical restrictions.
